# Information-incorporated gene network construction with FDR control

**DOI:** 10.1093/bioinformatics/btae125

**Published:** 2024-03-02

**Authors:** Hao Wang, Yumou Qiu, Hongqing Guo, Yanhai Yin, Peng Liu

**Affiliations:** Department of Statistics, Iowa State University, Ames, IA 50010, United States; Department of Statistics, Iowa State University, Ames, IA 50010, United States; Department of Genetics, Development and Cell Biology, Iowa State University, Ames, IA 50010, United States; Department of Genetics, Development and Cell Biology, Iowa State University, Ames, IA 50010, United States; Department of Statistics, Iowa State University, Ames, IA 50010, United States

## Abstract

**Motivation:**

Large-scale gene expression studies allow gene network construction to uncover associations among genes. To study direct associations among genes, partial correlation-based networks are preferred over marginal correlations. However, FDR control for partial correlation-based network construction is not well-studied. In addition, currently available partial correlation-based methods cannot take existing biological knowledge to help network construction while controlling FDR.

**Results:**

In this paper, we propose a method called Partial Correlation Graph with Information Incorporation (PCGII). PCGII estimates partial correlations between each pair of genes by regularized node-wise regression that can incorporate prior knowledge while controlling the effects of all other genes. It handles high-dimensional data where the number of genes can be much larger than the sample size and controls FDR at the same time. We compare PCGII with several existing approaches through extensive simulation studies and demonstrate that PCGII has better FDR control and higher power. We apply PCGII to a plant gene expression dataset where it recovers confirmed regulatory relationships and a hub node, as well as several direct associations that shed light on potential functional relationships in the system. We also introduce a method to supplement observed data with a pseudogene to apply PCGII when no prior information is available, which also allows checking FDR control and power for real data analysis.

**Availability and implementation:**

R package is freely available for download at https://cran.r-project.org/package=PCGII.

## 1 Introduction

The advent of microarray and RNA-sequencing technologies allows gene expression studies for the whole transcriptome and offers the opportunity to explore functional relationships among all genes using network analysis (Barabasi and Oltvai 2004, Boccaletti 2010, Cowen *et al.* 2017). The key to building a gene network involves identifying the connection (edges) among genes (nodes) to describe how genes are related. Various methods have been developed to construct gene networks. Some widely applied approaches, such as WGCNA (Zhang and Horvath 2005) and ARACNE (Margolin *et al.* 2006), apply marginal correlations that measure whether the expression levels of two genes simultaneously change in the same or opposite directions, regardless of other genes. Some approaches, such as Gaussian graphical models (GGMs), are based on conditional correlations that measure the correlations between genes while controlling for the effects of other genes. Consider a scenario where gene A inhibits gene B and gene C. Even without a direct interaction between B and C, the marginal association between B and C is positive due to the shared inhibitor, gene A. Such marginal correlations can be misleading in network analyses focusing on direct associations (Zuo *et al.* 2014). In contrast, conditional correlations account for confounding effects and identify connections between genes that others cannot explain. GGMs construct networks based on partial correlation, a type of conditional correlations, and can identify direct associations among genes. In addition, GGM offers advantages in terms of well-established statistical properties and computational efficiency. Hence, this paper focuses on networks built by GGMs.

Building gene networks by GGM faces a challenge arising from the extensive number of nodes. When the number of genes is much larger than the sample size, classical computations of GGM are not applicable. Sparse GGMs have been developed (Schäfer and Strimmer 2005, Meinshausen and Bühlmann 2006, Friedman *et al.* 2008, Peng *et al.* 2009) to identify the network in high-dimensional settings, but none of these methods control false discovery rate (FDR). Given the substantial number of possible connections that need to be evaluated, controlling FDR is essential to generate a reliable network while minimizing false positives. Recently, three approaches (Ren *et al.* 2015, Bernal *et al.* 2019, Qiu and Zhou 2020) have been proposed to recover a large-scale network while controlling FDR, but their performances need more investigation.

For well-studied organisms, some knowledge about direct associations among genes is available before constructing networks. Approaches capable of integrating such existing biological knowledge are particularly appealing. Benedetti *et al.* (2020) chooses network sparsity by comparing marginal correlation-based networks to the available prior knowledge. Yi *et al.* (2021) incorporates information into data-driven GGM to enhance power. Although these methods allow information incorporation in network analysis, they do not have a procedure to control FDR.

To the best of our knowledge, there are no available methods to build gene networks based on conditional correlations while incorporating biological information and controlling FDR at the same time. To fill the gap, we propose a method called Partial Correlation Graph with Information Incorporation (PCGII), which determines direct associations based on partial correlations, takes prior information while building gene networks, and controls FDR. We estimate partial correlations with estimated coefficients and residuals from regularized node-wise regressions. Given biological information, we leave out penalization on those known direct associations, resulting in more accurate coefficient estimates. We provide a de-biased estimator for partial correlations and propose a method to control FDR to recover nonzero partial correlations, leading to the constructed network. We demonstrate that PCGII controls FDR even when the prior information includes false positives, and it achieves higher power than the methods without utilizing prior biological information.

We apply PCGII to a plant study of the endoplasmic reticulum (ER) body formation-associated genes (Wang *et al.* 2017) and identify the confirmed regulatory relationship and a hub gene that functionally interacts with others. PCGII also discovers connections among some important genes, indicating more direct gene–gene associations related to ER body formation than currently known. We also develop and apply an analysis technique on an *Escherichia coli* dataset, illustrating network construction and evaluation when prior knowledge is not available.

This article is organized as follows. We describe our proposed PCGII procedure in Section 2. In Section 3, we present simulation studies under different network settings to compare the performance of PCGII with available methods that build partial correlation-based networks and control FDR. Results of applying PCGII to two real datasets are presented in Section 4 followed by a discussion in Section 5.

## 2 Materials and methods

Suppose a gene expression dataset has *p* genes and *n* samples. Let $Y_{i,j}$ denote the observed expression level of the *j*th gene from the *i*th sample, where i=1,…,n and j=1,…,p. Assume $Y_{i}=\left(Y_{i,1},\ldots ,Y_{i,p}\right)\prime$ to be independent and identically distributed (i.i.d.) random vectors with mean μ and covariance matrix $\Sigma ={\left(\sigma_{j_{1},j_{2}}\right)}_{p\times p}$. Let $\Omega =\Sigma^{-1}={\left(\omega_{j_{1},j_{2}}\right)}_{p\times p}$ be the corresponding precision matrix and $\rho_{j_{1},j_{2}}=-\omega_{j_{1},j_{2}}{\left(\omega_{j_{1},j_{1}}\omega_{j_{2},j_{2}}\right)}^{-1/2}$ be the partial correlation between the $j_{1}$th and $j_{2}$th nodes (genes).

### 2.1 Node-wise regression

Lemma 1 in Peng *et al.* (2009) established the connection between a partial correlation matrix and the variance-covariance matrix of the errors from *p* node-wide regressions. For each of the *p* variables, the node-wise regression of the $j_{1}$th variable takes the form

(1) $$\begin{array}{c} Y_{i,j_{1}}=\alpha_{j_{1},0}+\sum_{j_{2}\neq j_{1}}\alpha_{j_{1},j_{2}}Y_{i,j_{2}}+\xi_{i,j_{1}}, \end{array}$$

where $\xi_{i,j_{1}}$ is un-correlated with $Y_{i,j_{2}}$ for $j_{2}\neq j_{1}$. Let $V=(v_{j_{1},j_{2}})$ be the variance-covariance matrix of $\xi_{i}=\left(\xi_{i,1},\ldots ,\xi_{i,p}\right)\prime$. The partial correlation between $Y_{i,j_{1}}$ and $Y_{i,j_{2}}$ can be written as

(2) $$\begin{array}{c} \rho_{j_{1},j_{2}}=-\frac{v_{j_{1},j_{2}}}{\sqrt{v_{j_{1},j_{1}}\times v_{j_{2},j_{2}}}}. \end{array}$$

As the dimension of genes is typically high, lasso estimation can be used to fit the node-wise regressions in Equation (1) by the minimization program:

$$\begin{array}{c} \underset{\alpha_{j_{1}},\alpha_{j_{1},0}}{\text{argmin}}\frac{1}{n}\sum_{i=1}^{n}{\left(Y_{i,j_{1}}-\alpha_{j_{1},0}-\sum_{j_{2}\neq j_{1}}\alpha_{j_{1},j_{2}}Y_{i,j_{2}}\right)}^{2}+\lambda_{j}|\alpha_{j_{1}}{|}_{1}, \end{array}$$

where $\alpha_{j_{1}}=\left(\alpha_{j_{1},1},\ldots ,\alpha_{j_{1},p}\right)\prime$ with $\alpha_{j_{1},j_{1}}$ set as −1, and $\lambda_{j}$ is the penalty parameter (Liu 2013). Let ${\tilde{\alpha }}_{j_{1}}=\left({\tilde{\alpha }}_{j_{1},1},\ldots ,{\tilde{\alpha }}_{j_{1},p}\right)\prime$ be the lasso estimate of $\alpha_{j_{1}}$, and ${\hat{\xi }}_{i}=\left({\hat{\xi }}_{i,1},\ldots ,{\hat{\xi }}_{i,p}\right)\prime$ be the residuals from the lasso fit. Liu (2013) and Qiu and Zhou (2020) showed the sample covariance of residuals ${\left\{{\hat{\xi }}_{i}\right\}}_{i=1}^{n}$ is biased for estimating $v_{j_{1},j_{2}}$, and proposed the bias corrected estimator

(3) $$\begin{array}{c} {\tilde{v}}_{j_{1},j_{2}}=-\frac{1}{n}\sum_{i=1}^{n}\left({\hat{\xi }}_{i,j_{1}}{\hat{\xi }}_{i,j_{2}}+{\tilde{\alpha }}_{j_{1},j_{2}}{\hat{\xi }}_{i,j_{2}}^{2}+{\tilde{\alpha }}_{j_{2},j_{1}}{\hat{\xi }}_{i,j_{1}}^{2}\right). \end{array}$$

Note that, since ${\tilde{\alpha }}_{j,j}=-1$ by our definition, ${\tilde{v}}_{j,j}=\sum_{i=1}^{n}{\hat{\xi }}_{i,j}^{2}/n$ is the sample variance of the *j*th residual. The bias correction of estimating $v_{j_{1}j_{2}}$ is needed for $j_{1}\neq j_{2}$. From Equation (2), the node-wise regression estimate for partial correlation is ${\tilde{\rho }}_{j_{1},j_{2}}=-{\tilde{v}}_{j_{1},j_{2}}{\left({\tilde{v}}_{j_{1},j_{1}}{\tilde{v}}_{j_{2},j_{2}}\right)}^{-1/2}$.

### 2.2 Information incorporation

Qiu and Zhou (2020) showed that $\sqrt{n}{\tilde{\rho }}_{j_{1},j_{2}}$ converges to the standard normal distribution under suitable conditions if the true partial correlation between the $j_{1}$th and $j_{2}$th variable is zero. Therefore, we could use $\sqrt{n}{\tilde{\rho }}_{j_{1},j_{2}}$ as the test statistic to identify nonzero partial correlations among a set of genes. The CLEVEL procedure (Qiu and Zhou 2020) is built upon the estimate ${\tilde{\rho }}_{j_{1},j_{2}}$. However, this procedure fails to use prior knowledge of the network structure. Motivated by Wang *et al.* (2013), Benedetti *et al.* (2020), and Yi *et al.* (2021), we consider a more flexible scheme for gene network inference based on the node-wise regression approach, which can benefit from the prior biological knowledge.

Although the estimator ${\tilde{v}}_{j_{1},j_{2}}$ in Equation (3) is consistent to $v_{j_{1},j_{2}}$, as the $\ell_{1}$-norm penalty shrinks the lasso estimates toward zero, ${\tilde{\alpha }}_{j_{1},j_{2}}$ is biased for the true regression coefficient $\alpha_{j_{1},j_{2}}$. This would lead to an estimation error of the estimator ${\tilde{v}}_{j_{1},j_{2}}$ for the residual covariance. To overcome the bias issue of lasso estimation, if we know $\alpha_{j_{1},j_{2}}\neq 0$, we should not penalize $\alpha_{j_{1},j_{2}}$ in the node-wise regression in Equation (1). In practice, associations between some well-studied genes are often known. To utilize such biological information, we remove the penalty on the corresponding coefficients in the node-wise regressions. Specifically, given a set of known connections in a gene network $I=\{(j_{1},j_{2}):j_{1}\text{th},j_{2}\text{th genes are connected from prior knowledge}\}$, let $I_{j_{1}}=\{j_{2}:(j_{1},j_{2})\in I\}$ be the prior information set of connections with the $j_{1}$th gene. Let $I_{j}^{c}$ be the complement set of $I_{j}$. We propose to fit the node-wise regression of $Y_{i,j_{1}}$ without penalizing the coefficients from $I_{j_{1}}$. Let

$$\begin{array}{c} \begin{array}{c} {\hat{\alpha }}_{j_{1}}=\underset{\alpha_{j_{1}},\alpha_{j_{1},0}}{\text{argmin}}\frac{1}{n}\sum_{i=1}^{n}(Y_{i,j_{1}}\;-\;\alpha_{j_{1},0}\;-\sum_{j_{2}\neq j_{1}}\alpha_{j_{1},j_{2}}Y_{i,j_{2}}{)}^{2}\\ +\lambda_{j}\sum_{j_{2}\in I_{j_{1}}^{c}}|\alpha_{j_{1},j_{2}}| \end{array} \end{array}$$

be the lasso estimate with the incorporation of prior information, where ${\hat{\alpha }}_{j_{1}}=\left({\hat{\alpha }}_{j_{1},1},\ldots ,{\hat{\alpha }}_{j_{1},p}\right)\prime$ with ${\hat{\alpha }}_{j_{1},j_{1}}=-1$. Intuitively, if the true coefficient $\alpha_{j_{1},j_{2}}\neq 0$ and $j_{2}\in I_{j_{1}}$ is in the prior information set, the un-penalized estimate ${\hat{\alpha }}_{j_{1},j_{2}}$ should be more accurate than the lasso estimate ${\tilde{\alpha }}_{j_{1},j_{2}}$ without using the prior information. On the other hand, if $j_{2}\in I_{j_{1}}$ but $\alpha_{j_{1},j_{2}}=0$, the estimate ${\hat{\alpha }}_{j_{1},j_{2}}$ should still be close to zero.

We then use the prior information incorporated lasso estimator ${\hat{\alpha }}_{j_{1}}$ in Equation (3) to estimate the error covariance matrix V and the partial correlations. The proposed estimate takes the form

(4) $$\begin{array}{c} {\hat{\rho }}_{j_{1},j_{2}}=-{\hat{v}}_{j_{1},j_{2}}{\left({\hat{v}}_{j_{1},j_{1}}{\hat{v}}_{j_{2},j_{2}}\right)}^{-1/2},\;\text{where} \end{array}$$

(5) $$\begin{array}{c} {\hat{v}}_{j_{1},j_{2}}=-\frac{1}{n}\sum_{i=1}^{n}\left({\hat{\xi }}_{i,j_{1}}{\hat{\xi }}_{i,j_{2}}+{\hat{\alpha }}_{j_{1},j_{2}}{\hat{\xi }}_{i,j_{2}}^{2}+{\hat{\alpha }}_{j_{2},j_{1}}{\hat{\xi }}_{i,j_{1}}^{2}\right). \end{array}$$

The proposed estimate ${\hat{v}}_{j_{1},j_{2}}$ is in the same formulation as ${\tilde{v}}_{j_{1},j_{2}}$ in Equation (3), except the fully penalized lasso coefficient ${\tilde{\alpha }}_{j_{1},j_{2}}$ is replaced by the information incorporated coefficient ${\hat{\alpha }}_{j_{1},j_{2}}$. Note that we still use the residuals $\left\{{\hat{\xi }}_{i,j}\right\}$ from the fully penalized lasso regression. This is because the residuals from the information incorporated lasso regression could be small due to removing the penalties in the prior information set.

Under the conditions that data are Gaussian distributed, nonzero elements in each row of the precision matrix Ω are at a smaller order of $n^{1/2}{\left(\text{log}\;p\right)}^{-3/2}$ (a sparse precision matrix), and additionally the size of the prior information set for each variable is bounded, following the proof of Proposition 1 in Qiu and Zhou (2020), it can be shown that the information-incorporated partial correlation $\sqrt{n}{\hat{\rho }}_{j_{1},j_{2}}$ still converges to the standard normal distribution under the null hypothesis $\rho_{j_{1},j_{2}}=0$. Therefore, we can use the information-incorporated statistic $\sqrt{n}{\hat{\rho }}_{j_{1},j_{2}}$ to test for nonzero $\rho_{j_{1},j_{2}}$ and to construct the partial correlation graph. Note that the statistics for testing $\rho_{j_{1},j_{2}}=0$ based on ${\hat{p}}_{j_{1},j_{2}}$ and its Fisher’s transformation are asymptotically equivalent under the null hypothesis by Taylor’s expansion. For testing high-dimensional partial correlations, the classical methods for fixed-dimensional data are no longer applicable due to the rank deficiency of the sample covariance matrix. Our proposed estimator in Equation (4) is designed for high-dimensional data, corrects the biases induced by regularization, and can incorporate prior information.

### 2.3 FDR control

We consider two genes to be connected if their partial correlation is nonzero. Recovering the gene network amounts to testing the multiple hypotheses

(6) $$\begin{array}{c} H_{0}:\rho_{j_{1},j_{2}}=0\;\;v.s.\;H_{a}:\rho_{j_{1},j_{2}}\neq 0. \end{array}$$

Based on the proposed estimator ${\hat{\rho }}_{j_{1},j_{2}}$ from Equation (4), we propose a multiple testing procedure for the hypotheses (6) to identify nonzero partial correlations with FDR control. The false discovery proportion (FDP) is defined as the ratio of the number of false positives (FP) over the number of discovered connections (denoted as R), i.e. FDP=FP/R. In the context of a gene expression network, the false positives are the identified connections in the network but with the true partial correlation being zero. FDR is the expected value of FDP, which reflects the average proportion of false positives among discoveries over repeated experiments under the same condition.

Let Φ(·) be the cumulative distribution function of the standard normal distribution and $B(\tau )=2\;-\;2\Phi \{\tau \sqrt{\;\text{log}(p)}\}$. Given a threshold level τ, the candidate set of nonzero partial correlations is

(7) $$\begin{array}{c} D(\tau )=\{(j_{1},j_{2}):\sqrt{n}|{\hat{\rho }}_{j_{1},j_{2}}|>\tau \sqrt{\;\text{log}(p)}\}. \end{array}$$

Since $\sqrt{n}{\hat{\rho }}_{j_{1},j_{2}}$ converges to the standard normal if $\rho_{j_{1},j_{2}}=0$ as sample size increases, $\text{Pr}(\sqrt{n}|{\hat{\rho }}_{j_{1},j_{2}}|>\tau \sqrt{\;\text{log}(p)}|\rho_{j_{1},j_{2}}=0)\approx B(\tau )$. Therefore, the number of false positives in the set D(τ) is approximately $B(\tau )m_{0}$ which is bounded by $B(\tau )(p^{2}\;-\;p)$, where $m_{0}$ denote the number of true zero partial correlations among a set of *p* genes and is bounded by $p^{2}\;-\;p$ (the number of off-diagonal elements in a p×p matrix). To control FDR at the nominal level α, we could choose

(8) $$\begin{array}{c} {\hat{\tau }}_{\alpha }=\text{inf}\{\tau \in (0,2]:\frac{B(\tau )\times (p^{2}\;-\;p)}{\text{max}\{1,|D(\tau )|\}}\leq \alpha \}, \end{array}$$

where |D(τ)| denotes the size of the discovery set D(τ). The proposed PCGII procedure uses $D({\hat{\tau }}_{\alpha })$ as the significant set of nonzero partial correlations for the hypotheses (6).

The multiple testing procedure for PCGII follows Storey’s FDR control procedure (Storey 2002). Although the estimates $\left\{{\hat{\rho }}_{j_{1},j_{2}}\right\}$ are dependent, the validity of this FDR control procedure for high-dimensional sparse precision matrices and partial correlation matrices has been shown in Liu (2013) and Qiu and Zhou (2020). As we use $p^{2}\;-\;p$ as $m_{0}$, our approach is asymptotically equivalent to the Benjamini-Hochberg procedure (Benjamini and Hochberg 1995). The Benjamini-Yekutielli procedure (Benjamini and Yekutieli 2001) could be applied although the theoretical result of this procedure for testing high-dimensional partial correlation matrices has not been well studied.

## 3 Simulation studies

In this section, we evaluate the performance of our proposed PCGII procedure and compare it with three existing methods which control FDR but cannot take prior information: E-TEST (Bernal *et al.* 2019), F-GGM (Ren *et al.* 2015, Wang *et al.* 2016), and CLEVEL (Qiu and Zhou 2020). Other information-assisted methods such as Yi *et al.* (2021) cannot control FDR and are not included in the comparison.

### 3.1 Simulation settings

We simulated gene networks using three frequently used network structures: block diagonal, random nonzero locations, and scale-free.

- Block diagonal structure: The covariance matrix Σ of size p×p is block diagonal with blocks $B_{1},\ldots ,B_{H}$ on the main diagonal and zero elsewhere, where each block $B_{h}$ is compound-symmetric with size k×k, diagonal value $b_{h1}$ and off-diagonal value $b_{h2}$. The value of $b_{h1}$ was generated from Uniform (1,1.25), and $b_{h2}$ was generated from Uniform (0.3,0.5) for h=1,…,H.
- Random structure: The support of the precision matrix Ω was generated based on the Erdos-Renyi model (Erdős and Rényi 1960) by the R package *igraph* (Csardi and Nepusz 2006) with parameter η for the probability of drawing an edge between two arbitrary nodes.
- Scale-free structure: The support of the precision matrix Ω was generated based on the Barabasi-Albert model (Barabási 2009) by the R package *igraph* with parameter *e* for the density of edges.

For random and scale-free structures, the nonzero elements of the precision matrix in the support set were generated randomly from a uniform distribution with support (−0.5,−0.2)∪(0.2,0.5). To ensure positive-definiteness of Ω for those two structures, diagonal elements of Ω were equal to the row sums plus a small number, which we set as 0.1. For each network structure, we considered 2 different sparsity levels: the block size was k=4,8 for the block diagonal structure; the sparsity parameter was η=0.02,0.03 for the random structure; and the number of new edges generated in each step was e=1,2 for the scale-free structure. The dimension of networks was p=100 and 200. In total, we investigated 12 settings for all combinations of network structure, sparsity, and dimension. In addition, we simulated data using two moderate sample sizes n=60,80 and two small sample sizes n=10,15 for each of the 12 settings. The precision matrix Ω was fixed once generated for each setting, and 500 expression datasets of size *n* were simulated for both normal and count data types. For normal data, datasets were generated from a multivariate normal distribution with mean zero and covariance Σ. For count data, correlated data were drawn from a multivariate normal distribution with nonzero mean and covariance Σ, and discrete counts were obtained by rounding these continuous data, as suggested in Kruppa *et al.* (2016). In the main text, we primarily focus on the results for count data with moderate sample sizes. More simulation results for the other scenarios are presented in the supplementary materials. We chose the lasso penalty parameter in the node-wise regression as $\sqrt{2\;\text{log}(p/\sqrt{n})/n}$ following the suggestion in Wang (2015).

In practice, we do not expect the prior information to contain all true connections. To evaluate the effect of prior information size (PIS), we set the prior size to be 70% or 30% of the total number of true edges, denoted as PIS: 0.70 and PIS: 0.30, respectively. In addition, the prior information is not guaranteed to be 100% accurate in real applications. To mimic real situations when applying the PCGII method, we varied the prior accuracy (PA) to be 100% or 50%, where PA is defined as the proportion of true edges of all edges included in the prior information. Our proposed PCGII procedure was evaluated under the four combinations of two PIS levels and two PA levels for each setting.

### 3.2 Simulation results

To evaluate the performance of PCGII, E-TEST, F-GGM, and CLEVEL, we examined the ROC (receiver operating characteristic) curve, the Precision–Recall curve, and a variety of metrics, including empirical FDR, total power (recall), and excess power, which are calculated as the averages over 500 datasets for FDP, $\text{TP}/m_{1}$, and $\left(\text{TP}\;-\;{\text{TP}}_{\mathrm{prior}})/(m_{1}\;-\;{\text{TP}}_{\mathrm{prior}}\right)$, respectively, where $m_{1}$ denote the number of true nonzero partial correlations and ${\text{TP}}_{\mathrm{prior}}$ is the number of true positives in the prior set. Excess power is discussed in detail in Section 3.3.

**Figure 1.**
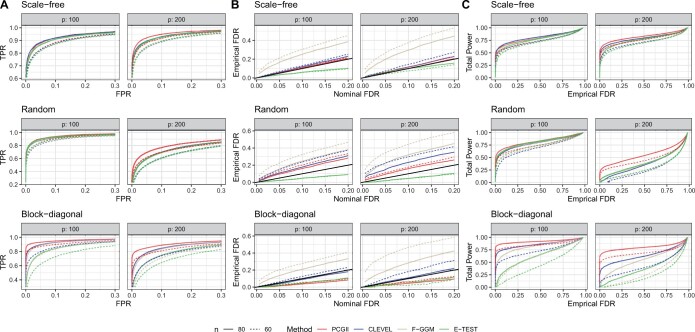
Simulation results for count data under sparse simulation settings. (A) ROC curves. (B) Empirical FDR. (C) Precision–Recall curves. Each panel includes three rows of plots under different network structures and dimensions. All presented network structures in this figure are sparse. Dashed and solid lines represent *n* = 60 and 80, respectively. Line colors indicate different approaches. The prior setting used for PCGII is PIS = 0.3 and PA = 100%.

The Benjamini-Hochberg method was applied to the *P*-values to control FDR for E-TEST and F-GGM. For CLEVEL and PCGII, we used the method described in Section 2.3 to control FDR. Figure 1 presents results for count data under sparse network conditions and Supplementary Fig. S1 presents the results under dense simulation settings, both for moderate sample sizes. Supplementary Figure S2 presents all results for normal data with moderate sample sizes. For small sample sizes, Supplementary Figs S3 and S4 exhibit results for count and normal data, respectively.

ROC curves plot the true positive rate (TPR) versus the false positive rate (FPR), where TPR is the proportion of true positives among the total number of nonzero partial correlations, and FPR is the proportion of false positives among the total number of zero partial correlations. ROC curves evaluate the ranking of true edges and nonedges for each method, and higher curves indicate better performances. ROC curves in panel (A) of Fig. 1 and Supplementary Figs S1–S4 show that PCGII is the best performer or among the best performers for all settings. The other three approaches have comparable performance for the scale-free and random network structures. For the block-diagonal structure, CLEVEL is substantially better than E-TEST and F-GGM, and PCGII is substantially better than CLEVEL.

We also evaluate the FDR control of each method by plotting the empirical FDR versus the nominal level. Lines close to the y=x line (solid black line in the plots) indicate desired FDR control. As shown in panel (B) of Fig. 1 and Supplementary Figs S1–S4, for all simulation settings, F-GGM is very liberal and cannot control FDR to the nominal level, while E-TEST tends to be conservative. For dense settings with moderate sample sizes, the empirical FDR of PCGII is very close to the nominal level, indicating that PCGII controls FDR to the desired level. For sparse settings with moderate sample sizes as shown in Fig. 1 and Supplementary Fig. S2, CLEVEL and PCGII control FDR very well under the scale-free network structure. Under the random structure, both CLEVEL and PCGII are liberal but to a much lesser degree than F-GGM. Under the block-diagonal structure, PCGII is a little conservative, while CLEVEL controls FDR closer to the nominal level. Results for small sample sizes in Supplementary Figs S3 and S4 show that the FDR control tends to be less effective compared to settings with moderate sample sizes for all methods, while PCGII is still the best method with empirical FDR closest to the nominal level. Taking all scenarios into consideration, PCGII provides the best FDR control overall.

Recall (statistical power) and Precision (FDR control) are two compromising aspects of a statistical test. We plot Precision–Recall curves in panel (C) of Fig. 1 and Supplementary Figs S1–S4. For the same level of Precision, a method achieving higher power than others would be the best-performing method. As shown in panel (C) of Fig. 1 and Supplementary Fig. S1, PCGII achieves the highest power across the whole range of empirical FDR for all settings except for the low dimension setting under scale-free structure for the sparse network and moderate sample sizes, where PCGII, CLEVEL, and F-GGM achieve comparable power that is higher than E-TEST. For all other simulation settings, Supplementary Figs S2–S4 demonstrate that PCGII always achieves the highest power among all methods, and the improvement over the second-best performing method is quite substantial.

Overall, the simulation results show that PCGII provides a better ranking of edges, demonstrates desired FDR control, and achieves higher total power than other methods. PCGII can detect a larger number of true connections while maintaining the FDR controlled to the desired level, providing a more reliable network.

### 3.3 Excess power

The simulation results in Section 3.2 show that PCGII does benefit from incorporating prior information compared with CLEVEL. In this section, we evaluate the advantages of incorporating prior connections and the capability of PCGII to identify *additional edges besides those in the prior information*. To do this, we computed the “excess power” in detecting the remaining connections after excluding edges in the prior set by taking the average of $\left(\text{TP}\;-\;{\text{TP}}_{\mathrm{prior}})/(m_{1}\;-\;{\text{TP}}_{\mathrm{prior}}\right)$ over 500 simulated datasets for each simulation setting on count data with moderate sample sizes. Such evaluation provides insights into PCGII’s performance in identifying unknown connections in the network, offering a comprehensive understanding of its overall performance in network reconstruction. The excess power of the other three approaches was computed similarly.

**Figure 2.**
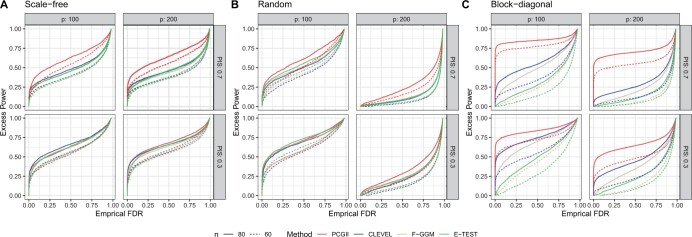
Simulation results of excess power for count data under sparse simulation settings. Excess power under different combinations of network dimensions (*p*), and prior information size (PIS). Prior accuracy (PA) is 100%. Panels (A–C) correspond to scale-free, random, and block-diagonal structures, respectively. All presented network structures in this figure are sparse. Dashed and solid lines represent *n* = 60 and 80. Line colors indicate different approaches.

Figure 2 presents the results of excess power versus empirical FDR for sparse simulation settings. When prior information is 100% accurate (i.e. PA = 1, as in Fig. 2) and the prior information includes 70% of true edges (i.e. PIS = 0.7), PCGII achieves the highest excess power than other approaches, indicating PCGII detects more additional true edges outside of the prior set than other methods. For the block-diagonal structure, the difference between PCGII and CLEVEL, the next best-performing method, is quite large. When prior information is still 100% accurate (PA = 1) but the prior information includes only 30% of true edges (PIS = 0.3), PCGII achieves the highest excess in most cases but is the second best-performing method for the scale-free setting when p=100. The results indicate that PCGII’s excess power increases with the inclusion of more correct information. Such benefits from incorporation are more pronounced as the dimension of networks increases. The results from dense networks lead to the same conclusion (Supplementary Figs S5–S7).

When half of the edges incorporated are false connections, i.e. PA = 0.5, PCGII is no longer the method with the highest excess power for low levels of empirical FDR (Supplementary Figs S5–S7). When prior information includes a large proportion of false information, the drop in PCGII’s performance is because PCGII prioritizes all incorporated connections regardless of their accuracy, and incorporating false information inflates empirical FDR. Hence, we recommend practitioners only incorporate reliable information in the prior set to improve the prior accuracy and take advantage of PCGII.

## 4 Analysis of real datasets

### 4.1 FERONIA network analysis

FERONIA is a receptor kinase that regulates plant growth and development, stress responses, and reproduction. It has been suggested that FERONIA regulates endoplasmic reticulum (ER) body formation through the transcription factor NAI1 (Wang *et al.* 2022). Scientists are interested in learning (i) whether other transcription factors are involved in ER body formation mediated by FERONIA, and (ii) whether ER body-related genes are directly associated with each other and if so, how FERONIA is involved in such direct associations. To address these questions, we built the gene network of FERONIA and a list of 17 known ER body-associated genes (Wang *et al.* 2017) based on the gene expression study in Arabidopsis (Wang *et al.* 2022). This study included two genotypes (wild type and *fer-4* loss-of-function mutant) with three biological replicates for each genotype. We centered data by genotype before network analysis to control the treatment effect. When applying PCGII, we included the FERONIA-NAI1 connection and five confirmed functional connections between transcription factor NAI1 and its target genes *JAL34*, *NAI2*, *BGLU23*, *MEB1*, and *GLL23* (Wang *et al.* 2017). In total, six connections were included in the prior set for PCGII as shown in panel (a) of Supplementary Fig. S8.

**Figure 3.**
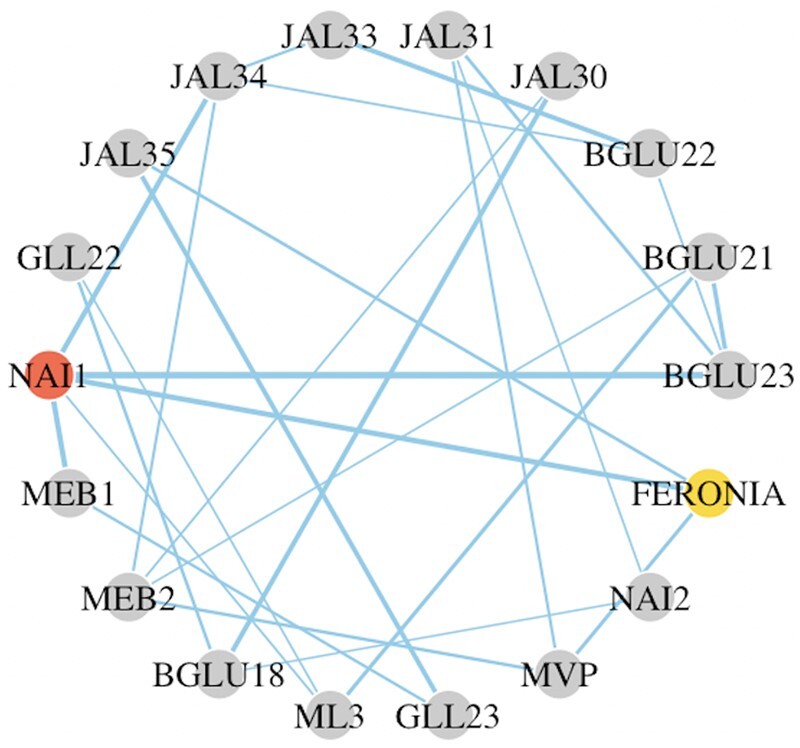
Re-constructed network for FERONIA by PCGII.

PCGII, CLEVEL, E-TEST, and F-GGM were applied to construct the network, and they identified 28 (PCGII), 28 (CLEVEL), 0 (E-TEST), and 36 (F-GGM) edges, respectively, when FDR was controlled at 0.05. See Fig. 3 and Supplementary Fig. S9. Similar to the performance in simulation studies, E-TEST was very conservative and did not recover any connections in this network.

Comparing the constructed networks with confirmed functional connections, these methods respectively affirmed 5 (PCGII), 1 (CLEVEL), and 0 (F-GGM) out of the 6 priorly confirmed connections. CLEVEL confirmed one validated connection (FERONIA-NAI1) (Wang *et al.* 2022) without incorporating information and PCGII recovered most of the confirmed connections benefiting from information incorporation. PCGII and CLEVEL both detected another edge (NAI1-ML3) that was not in the prior. We later found that this connection was a confirmed functional relationship in Wang *et al.* (2022). PCGII also uniquely detected FERONIA-JAL35 and FERONIA-MVP connections. Because jacalin-related lectins (JALs) regulate the size of ER body-type β-glucosidase complexes (Nagano *et al.* 2008), our modeling suggests that additional transcription factor(s), possibly regulated by FERONIA, may function to mediate FERONIA-JAL35/MVP connections. This hypothesis can be examined by future biological experiments.

PCGII, CLEVEL, and F-GGM shared 17 common edges among the ER body-associated genes [see panel (b) of Supplementary Fig. S8]. By analyzing the dataset presented in Zander *et al.* (2020), we found that 8 out of the 17 common edges can be potentially explained by the corresponding genes’ co-expression with the transcription factor MYC2, which was not included in the network construction. MYC2 is an established phosphorylation target of FERONIA (Guo *et al.* 2018) and mediates jasmonic acid signaling to control ER body formation (Wang *et al.* 2017). So our results also suggest a role of MYC2 in ER body related gene expression.

### 4.2 Network construction when no prior information is available

In practice, there are gene expression studies with no prior information on gene–gene direct associations. In this subsection, we illustrate how to apply PCGII to such studies by introducing a “pseudogene” that has connections with some of the originally observed genes and using these connections as prior information. More specifically, we construct a supplemented dataset by generating ${\tilde{Y}}_{i,j}=Y_{i,j}+a_{j}Z_{i}$ for i=1,…,n and j=1,…,p, where $Y_{i,j}$ denote the original gene expression data for the *i*th sample and *j*th gene, $a_{j}$ is a coefficient that controls the connection between the pseudogene and the original set of genes, and $Z_{1},\ldots ,Z_{n}$ are the generated “expression levels” of the pseudogene and are i.i.d. from the standard normal distribution. Denote the supplemented data by ${\tilde{Y}}_{i}={\left({\tilde{Y}}_{i,1},\ldots ,{\tilde{Y}}_{i,p},Z_{i}\right)}^{\prime }$. It can be shown that the upper p×p block $\tilde{\Psi }$ of the partial correlation matrix of the supplemented data ${\tilde{Y}}_{i}$ is the same as the partial correlation matrix of the original data. Furthermore, the true partial correlation between $Y_{i,j}$ and $Z_{i}$ is determined by the coefficients $a_{j}$s. If $a_{j}\neq 0$, the partial correlation between the pseudogene and the *j*th gene is nonzero and there is an edge between them in the network. A more detailed description of the method is presented in Supplementary Section S4.1.

We applied such a method to an *E.coli* dataset obtained by Schmidt-Heck *et al.* (2004) to study the stress response during the production of recombinant protein. The dataset comprised 102 genes measured at 9 time points. We conducted network analysis on the original data as suggested by the previous studies (Schäfer and Strimmer 2005, Bernal *et al.* 2019). No prior information is available for this dataset.

We applied E-TEST, F-GGM, CLEVEL, and PCGII to the supplemented data $\left\{{\tilde{Y}}_{i}\right\}$ that was constructed with 20 nonzero $a_{j}$s for 20 randomly selected genes. Supplementary Section S4.2 of Supplementary Materials presents data supplementation and analysis details. We first evaluate the performance of four approaches in recovering the network involving the pseudogene. Table 1 shows the numbers of total discovered connections, discovered true connections, and discovered false connections, respectively. PCGII identified 19 out of 20 true edges and none of the null edges, providing high power with no type I errors. The other three approaches all discovered some false connections while identifying fewer true connections.

**Table 1.** Results on *E.coli* dataset.

Method	No. of total discoveries	No. of true discoveries	No. of false discoveries
PCGII	19	19	0
CLEVEL	7	3	2
E-TEST	10	3	2
F-GGM	13	2	4

We next report the identified connections of the upper p×p block $\tilde{\Psi }$, which is the same as the partial correlation matrix of the original data. When controlling FDR at 0.05, E-TEST exhibited a conservative nature, identifying only 8 edges among 102 nodes. Conversely, F-GGM detected a large number of edges (4342), potentially compromising the interpretability and meaningfulness of the network. In contrast, CLEVEL and PCGII discovered 245 and 240 edges. The varied performances aligned with our findings in simulation studies. Analysis of the original data without the pseudogene led to 4356, 9, and 245 detected edges for F-GGM, E-TEST, and CLEVEL, respectively, which is highly consistent with the analysis of the supplemented dataset (Supplementary Fig. S10). This indicates that supplementing the observed data with the pseudogene did not seem to change the main results of the other methods.

## 5 Discussion

In this article, we develop a new approach called PCGII to construct gene networks by incorporating biological information into regularized node-wise regression when estimating partial correlations. The proposed approach allows prior biological knowledge to assist network construction, which is not feasible for other GGM-based network inference methods. In addition, PCGII controls FDR much better than other methods, which can be either too conservative (E-TEST) or too liberal (F-GGM). Benefiting from the prior information, PCGII exhibits enhanced signal detection ability in both simulation and case studies.

For well-studied organisms, some gene–gene direct associations have been validated by independent experiments, establishing highly reliable prior information. Such prior information provides confirmed network connections. PCGII effectively utilizes such information to reconstruct the network. Our analysis of excess power shows that when prior information is accurate, prior information incorporation not only maintains those confirmed connections but also helps identify new connections not in the prior set, benefiting the construction of the whole network.

In some cases, it is possible that some known direct associations may be experimental artifacts and are not as reliable. To explore the impact of prior information accuracy and size on PCGII, we conducted a sensitivity analysis. The results are presented in Supplementary Section S5. In general, PCGII’s power positively correlates with the inclusion of correct prior information. However, incorporating false information could lead to an inflation of the empirical FDR. As a result, we strongly recommend incorporating only high-confidence information in real data applications.

Our proposed PCGII can handle large and complex network structures with efficient computation. For a dataset with 200 nodes, it takes about 1 s on a MacBook Pro with Apple M1, 10-core CPU, 24-core GPU, 14 GB of RAM, and 1 TB SSD. PCGII utilizes penalized node-wise regression and can handle high-dimensional data. In addition, PCGII can be used not only to analyze transcriptomic data but also to analyze other omics data such as metabolomics, proteomics, and microbiome data. The R package PCGII is freely available for download at https://cran.r-project.org/package=PCGII.

## Supplementary Material

btae125_Supplementary_Data

## Data Availability

Publicly available datasets were analyzed in this study. This data can be found here: Gene Expression Omnibus (https://www.ncbi.nlm.nih.gov/geo/) under series GSE143634.
