# Supplementary material for: Information-incorporated gene network construction with FDR control
Source: Bioinformatics. 2024 Mar 2;40(3):btae125. doi: 10.1093/bioinformatics/btae125 (PMC10937901; doi:10.1093/bioinformatics/btae125)
Supplement: btae125_Supplementary_Data [file btae125_supplementary_data.pdf]

Supplementary Materials for Information-incorporated Gene Network Construction with FDR Control

Hao Wang
Department of Statistics
Iowa State University
Ames, IA, USA
halewang@iastate.edu

Yumou Qiu*
Department of Statistics
Iowa State University
Ames, IA, USA
yumouqiu@iastate.edu

Hongqing Guo
Department of Genetics, Development and Cell Biology
Iowa State University
Ames, IA, USA
hguo@iastate.edu

Yanhai Yin
Department of Genetics, Development and Cell Biology
Iowa State University
Ames, IA, USA
yin@iastate.edu

Peng Liu*
Department of Statistics
Iowa State University
Ames, IA, USA
pliu@iastate.edu

Contents

S-1 Supplementary Results for Simulation Studies **3**
S-1.1 Simulation Results of Moderate Sample Sizes 3
S-1.2 Simulation Results of Small Sample Sizes 5

S-2 Supplementary Results for Analysis of Excess Power **7**

S-3 Supplementary Results for Plant Gene Network Analysis **10**

S-4 Supplementary Results for Supplemented Data Analysis on *E. coli* Dataset **11**
S-4.1 Methodology 11
S-4.2 Application on *E. coli* Dataset 11

S-5 Supplementary Results for Sensitivity Analysis **14**

List of Figures

S-1 Simulation Results for Count Data under Dense Network Settings with Moderate Sample Sizes 3
S-2 Simulation Results for Normal Data with Moderate Sample Sizes 4
S-3 Simulation Results for Count Data with Small Sample Sizes 5
S-4 Simulation Results for Normal Data with Small Sample Sizes 6

S-5 Excess Power for Count Data under The Scale-free Structure 7
S-6 Excess Power for Count Data under The Random Structure 8
S-7 Excess Power for Count Data under The Block-diagonal Structure 9
S-8 Supplementary Figures for FERONIA Network Analysis 10
S-9 Reconstructed FERONIA Networks by CLEVEL and F-GGM 10
S-10 Venn Diagrams of Reconstructed *E. coli* Networks 13
S-11 Performance of PCGII across Different Settings of Prior Information under The Scale-free Structure 15
S-12 Performance of PCGII across Different Settings of Prior Information under The Random Structure 16
S-13 Performance of PCGII across Different Settings of Prior Information under The Block Diagonal Structure 17

List of Tables

S-1 Summary of Supplemented Data Analysis on *E. coli* Dataset. 12

S-1 Supplementary Results for Simulation Studies

S-1.1 Simulation Results of Moderate Sample Sizes

This subsection presents additional simulation results on the performance of E-TEST, F-GGM, CLEVEL, and PCGII. Supplementary Figure S-1 is the result for count data under dense network settings with moderate sample sizes in a way presented similar to Figure 1 in the main manuscript. The advantage of PCGII becomes increasingly evident as the network structure grows more intricate. To sum up, Figure 1 and Supplementary Figure S-1 together illustrate that E-TEST cannot achieve high power as it is very conservative after FDR control, while F-GGM can detect more edges but fails to control FDR, resulting in a great number of false edges. CLEVEL shows effective FDR control, and PCGII as a power-enhancing alternative to CLEVEL can detect a larger number of true edges while maintaining the FDR controlled to the desired level.

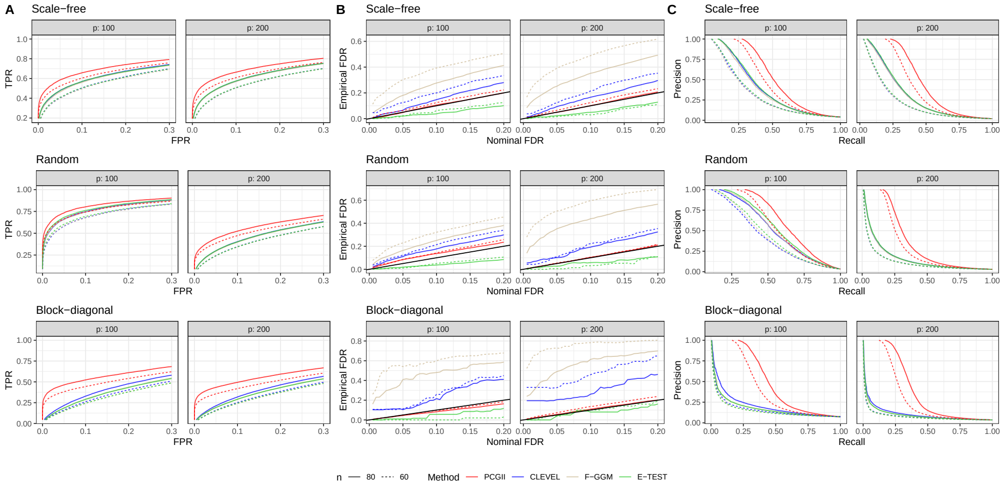

Figure S-1: Simulation Results for Count Data under Dense Network Settings with Moderate Sample Sizes.

Panel (A): ROC curves. Panel (B): False Discovery Rate. Panel (C): Precision-Recall curves. Each panel includes three rows of plots under different network structures and dimensions. Colors distinguish approaches. All presented network structures are dense. Dashed and solid lines represent $n = 60$ and $n = 80$, respectively. Line colors indicate different approaches. The prior setting used for PCGII is PIS=.3 and PA=1.

Supplementary Figure S-2 presents all results for normal data with moderate sample sizes. It shows the results are highly consistent with those for count data (Figure 1 and Supplementary Figure S-1) and suggests that four methods are robust to count data.

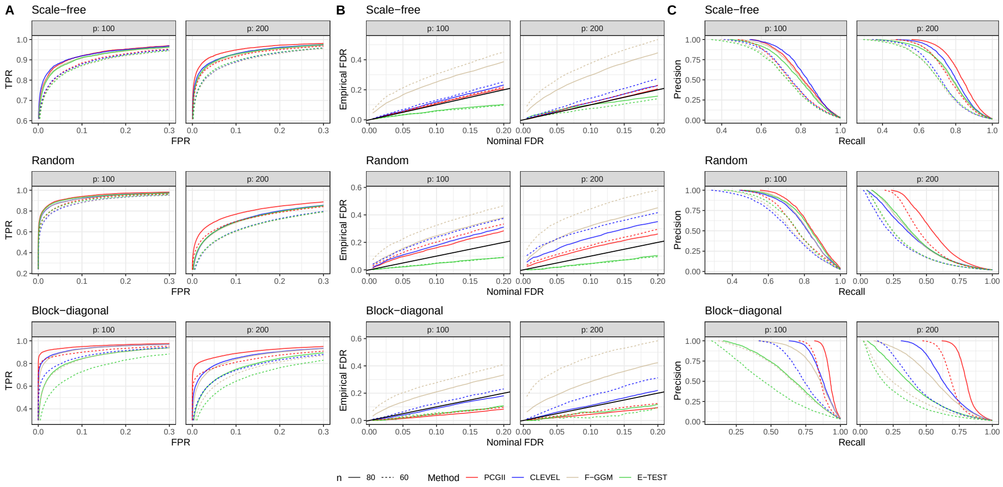

(a) Sparse Networks

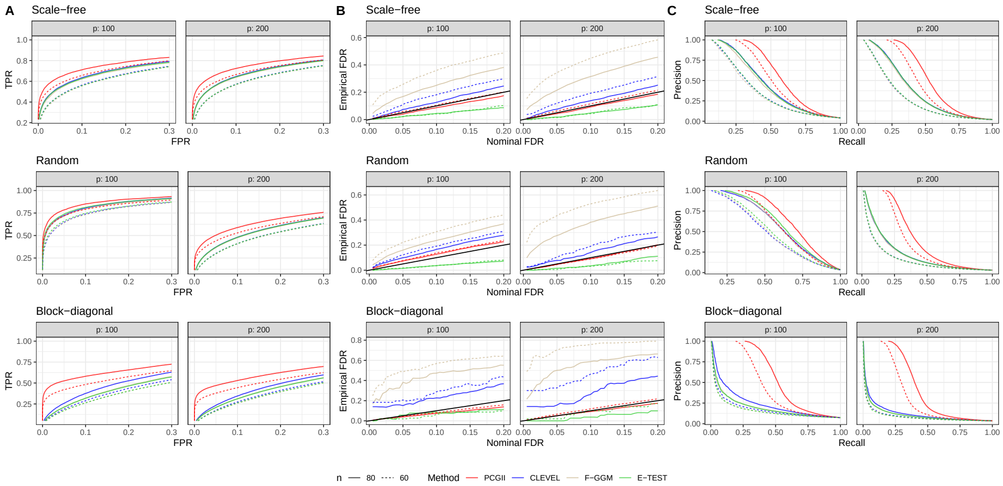

(b) Dense Networks

Figure S-2: Simulation Results for Normal Data with Moderate Sample Sizes.
For both sub-figures (a) and (b), panels are listed as follows. Panel (A): ROC curves. Panel (B): False Discovery Rate. Panel (C): Precision-Recall curves. Each panel includes three rows of plots under different network structures and dimensions. Colors distinguish approaches. All presented network structures are dense. Dashed and solid lines represent $n = 60$ and $n = 80$, respectively. Line colors indicate different approaches. The prior setting used for PCGII is PIS=.3 and PA=1.

S-1.2 Simulation Results of Small Sample Sizes

This subsection presents simulation results for both count and normal data with small sample sizes that may occur in some real studies. Supplementary Figures S-3 and S-4 are the results for count data and normal data respectively. The relative rankings of different methods stay the same, although power and FDR control are worse with small sample sizes than moderate sample sizes for all methods.

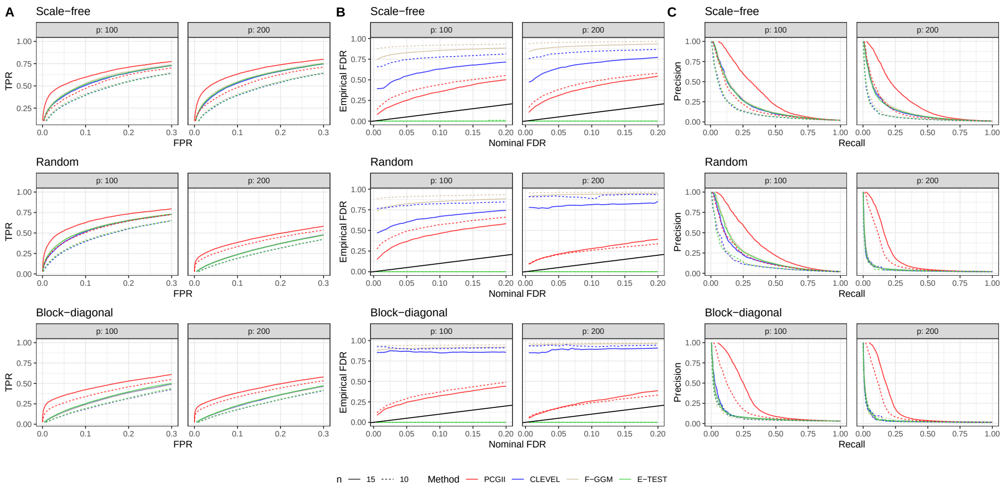

(a) Sparse Networks

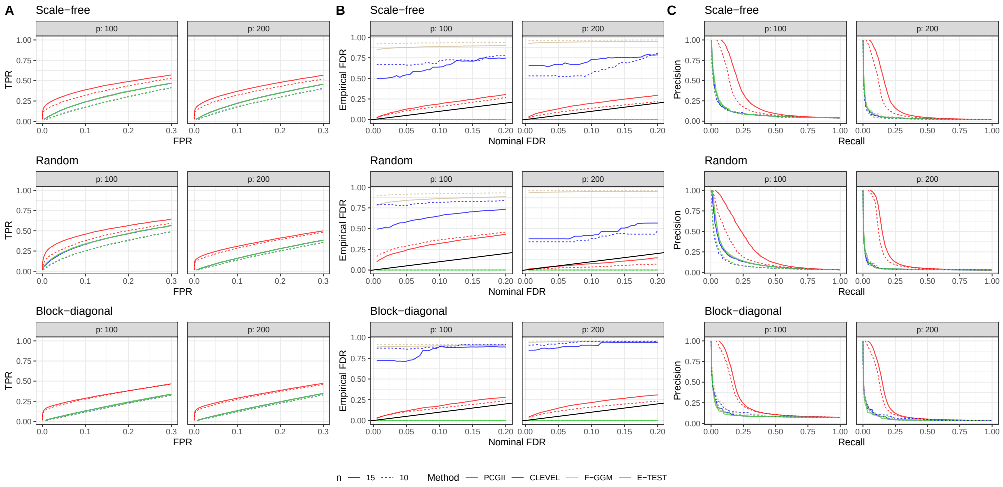

(b) Dense Networks

Figure S-3: Simulation Results for Count Data with Small Sample Sizes.
For both sub-figures (a) and (b), panels are listed as follows. Panel (A): ROC curves. Panel (B): False Discovery Rate. Panel (C): Precision-Recall curves. Each panel includes three rows of plots under different network structures and dimensions. Colors distinguish approaches. All presented network structures are dense. Dashed and solid lines represent $n = 10$ and $n = 15$, respectively. Line colors indicate different approaches. The prior setting used for PCGII is PIS=.3 and PA=1.

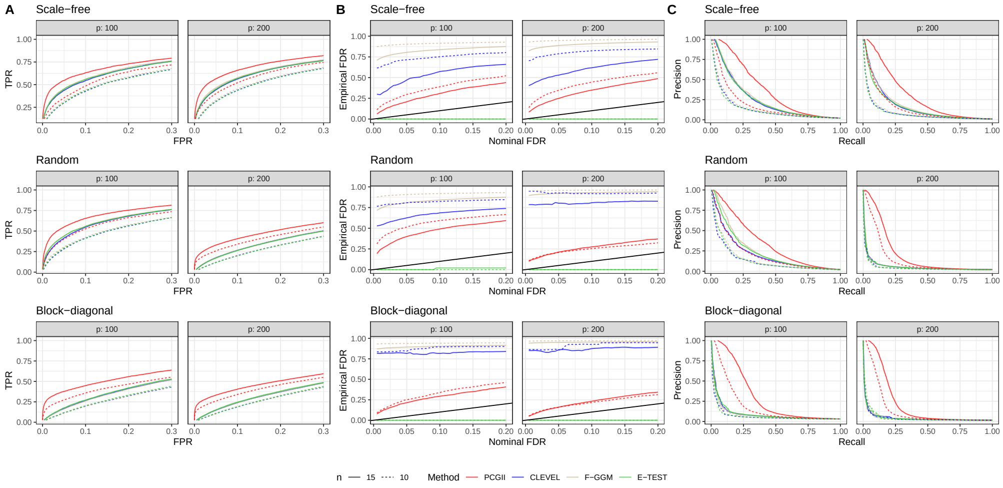

(a) Sparse Networks

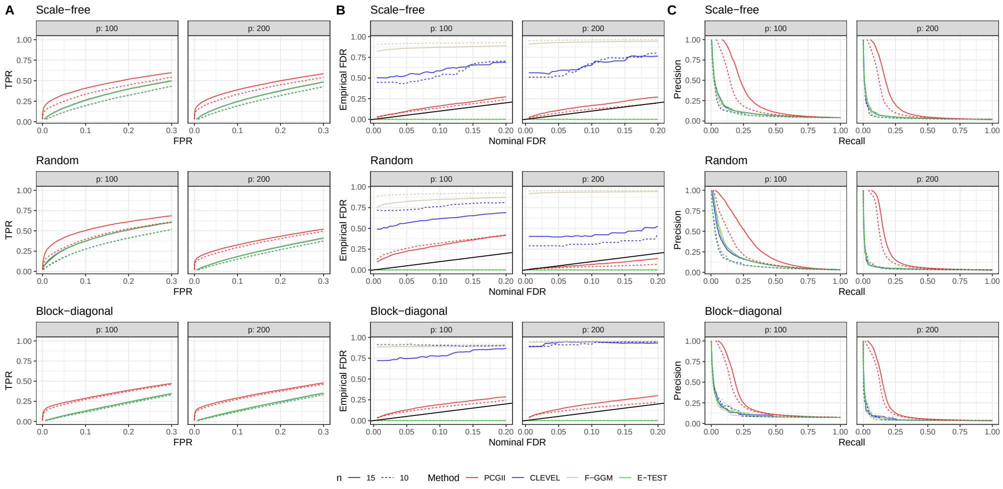

(b) Dense Networks

Figure S-4: Simulation Results for Normal Data with Small Sample Sizes.
For both sub-figures (a) and (b), panels are listed as follows. Panel (A): ROC curves. Panel (B): False Discovery Rate. Panel (C): Precision-Recall curves. Each panel includes three rows of plots under different network structures and dimensions. Colors distinguish approaches. All presented network structures are dense. Dashed and solid lines represent $n = 10$ and $n = 15$, respectively. Line colors indicate different approaches. The prior setting used for PCGII is PIS=.3 and PA=1.

S-2 Supplementary Results for Analysis of Excess Power

This section presents supplementary figures of excess power, defined in Section 3 of the main manuscript. Supplementary Figures S-5, S-6, and S-7 show results for count data with moderate sample sizes obtained under the scale-free, random and block-diagonal structures, respectively.

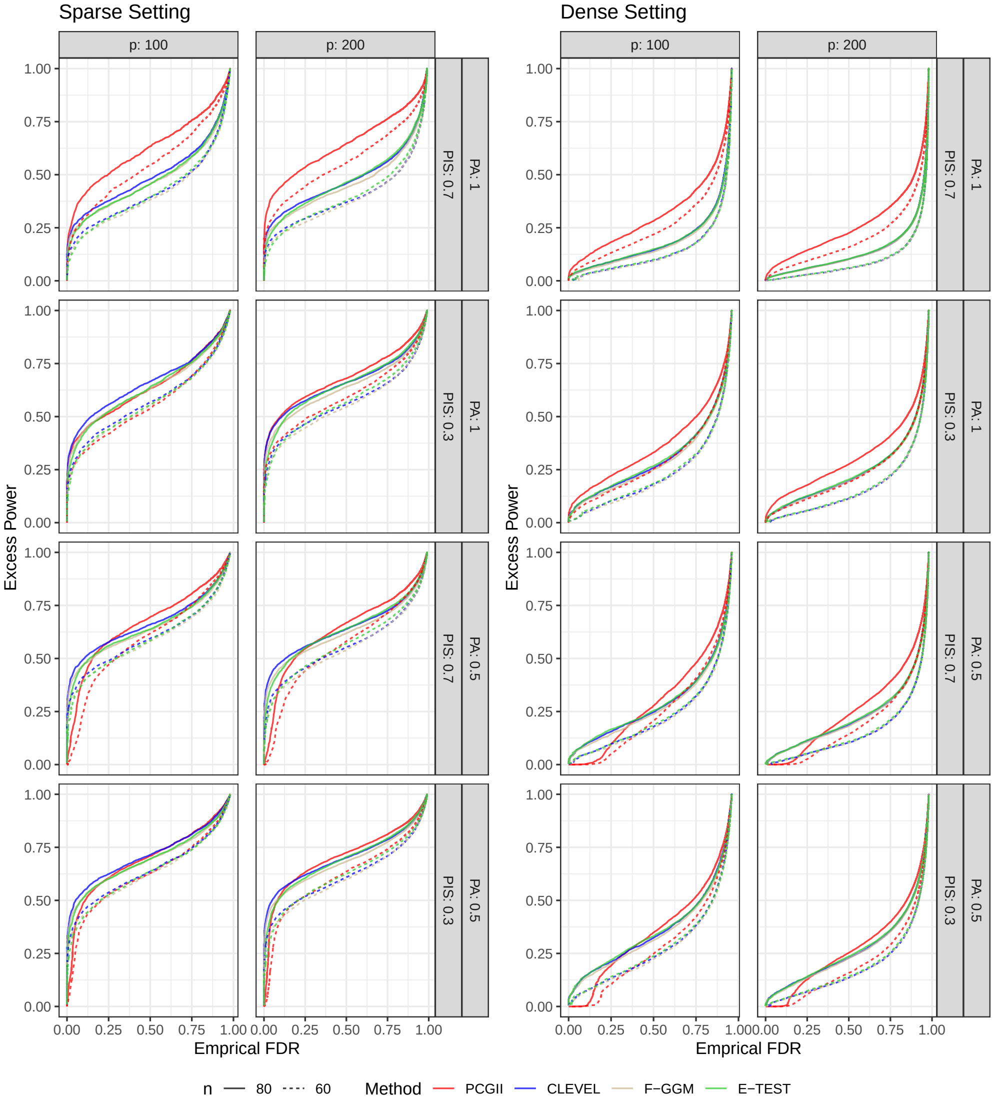

Figure S-5: Excess Power for Count Data under The Scale-free Structure.
Excess power versus empirical FDR curves under different combinations of p, η, PIS and PA. Dashed and solid lines represent n = 60 and 80, respectively. Line color indicates different methods.

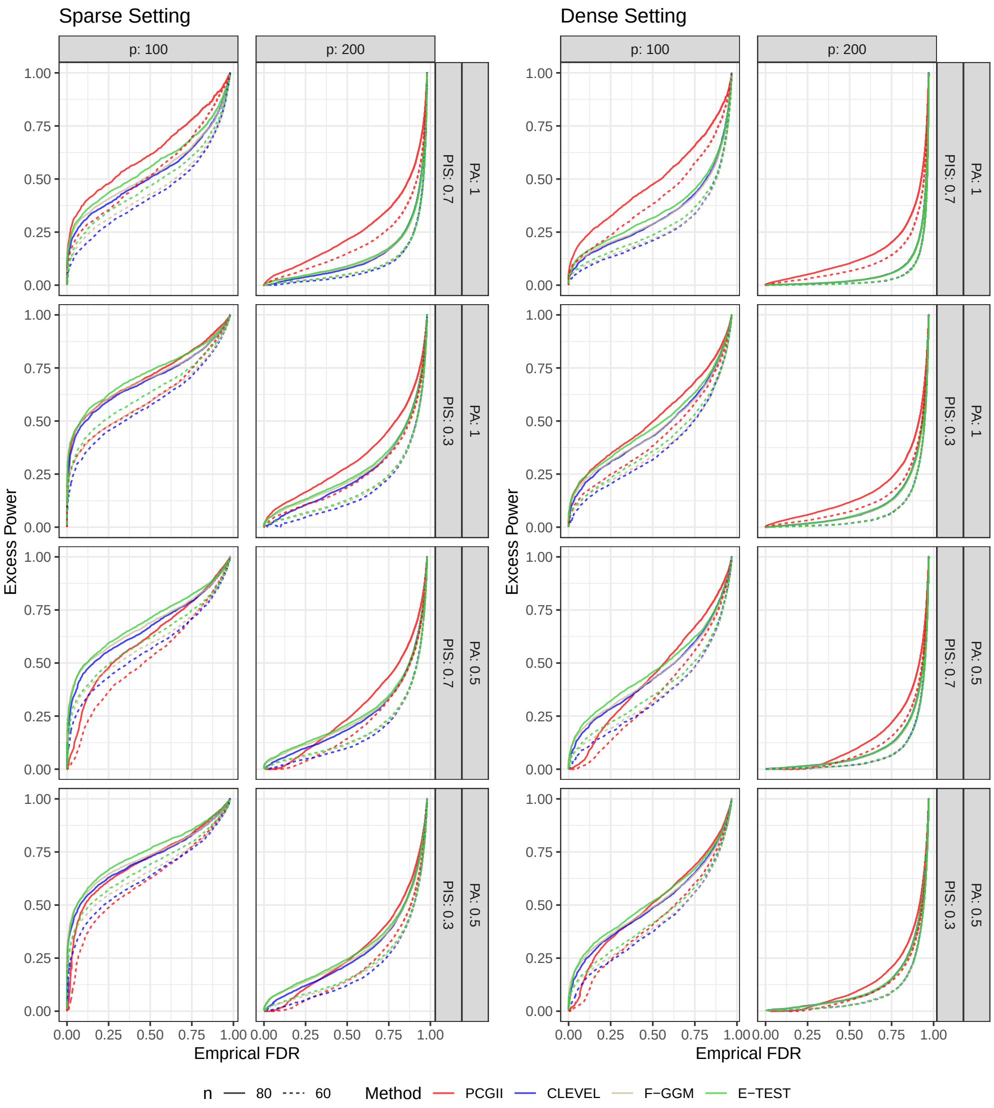

Figure S-6: Excess Power for Count Data under The Random Structure.
Excess power versus empirical FDR curves under different combinations of p, η, PIS and PA. Dashed and solid lines represent n = 60 and 80, respectively. Line color indicates different methods.

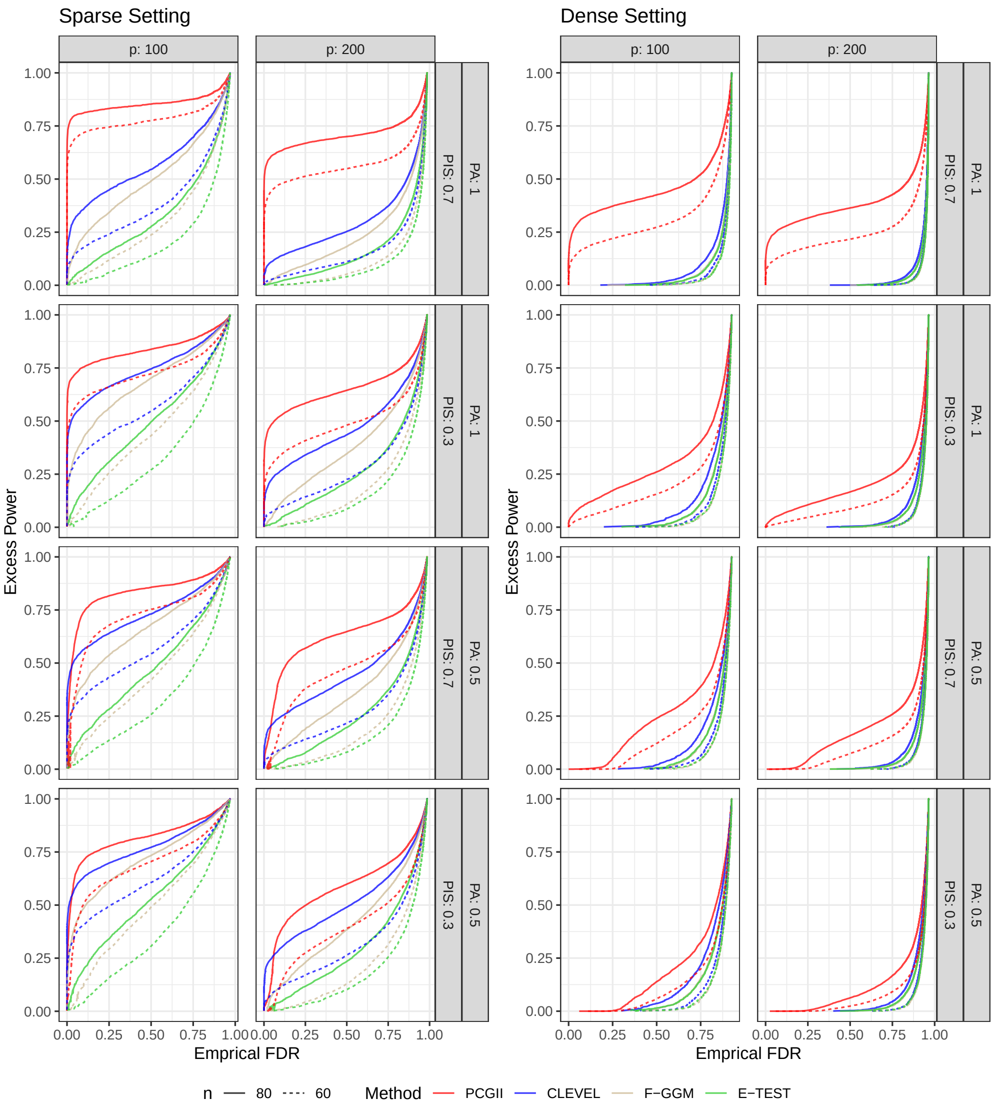

Figure S-7: Excess Power for Count Data under The Block-diagonal Structure.
Excess power versus empirical FDR curves under different combinations of p, k, PIS and PA. Dashed and solid lines represent $n = 60$ and 80, respectively. Line color indicates different methods.

S-3 Supplementary Results for Plant Gene Network Analysis

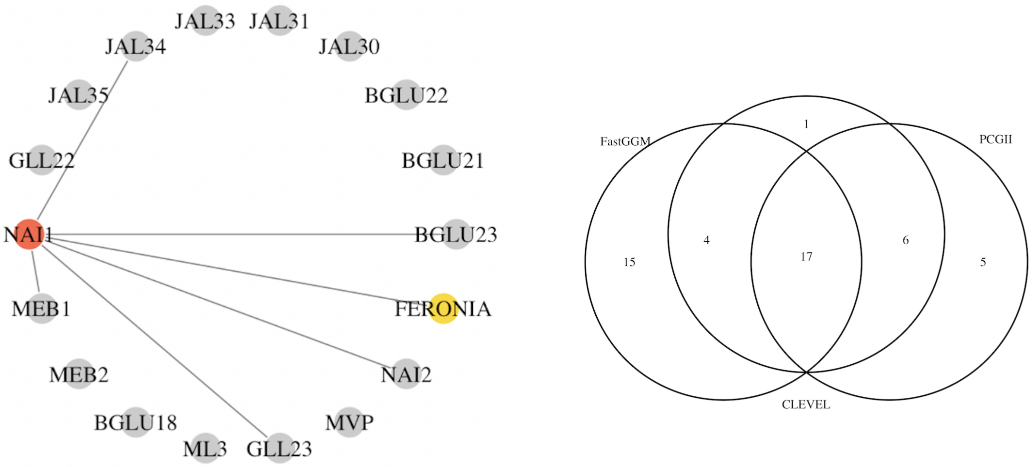

(a) (b)

Figure S-8: Supplementary Figures for FERONIA Network Analysis.
(a) Prior network used for PCGII; (b) Venn diagram: common connections between networks constructed by PCGII, CLEVEL, and F-GGM.

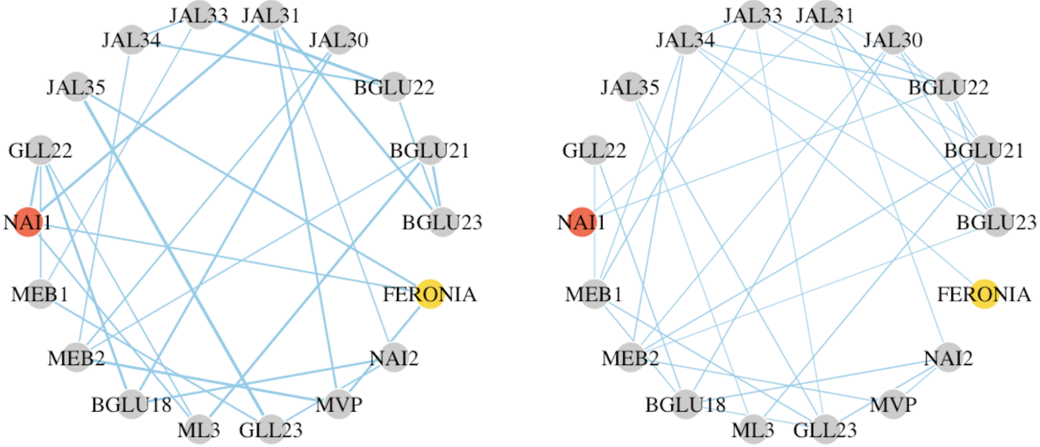

(a) (b)

Figure S-9: Reconstructed FERONIA Networks by CLEVEL and F-GGM.
(a) CLEVEL; (b) F-GGM. Blue and red edges indicate positive and negative partial correlations, respectively.

S-4 Supplementary Results for Supplemented Data Analysis on *E. coli* Dataset

S-4.1 Methodology

When prior knowledge of gene-gene associations is not available, incorporating information can be challenging. We propose supplementing the original network by including a pseudogene and analyzing the supplemented data. Connections between the pseudogene and the original set of genes will be used as prior knowledge. In addition, this supplemented information can be used to evaluate a method's performance.

Suppose a pseudogene connects with a subset of p nodes under the original study. Then based on the observed dataset $\{Y_{i,j}\}$, we can generate a supplemented dataset $\{\tilde{\mathbf{Y}}_i\}$, where $\tilde{\mathbf{Y}}_i = (\tilde{Y}_{i,1}, ..., \tilde{Y}_{i,p}, Z_i)'$, $\tilde{Y}_{i,j} = Y_{i,j} + a_j Z_i$, and $Z_1, ..., Z_n$ are i.i.d. from standard normal distribution. It is important to note the difference between $Y_{i,j}$ and $\tilde{Y}_{i,j}$, where the former is a sample of the jth node under the original $p \times p$ network but the latter is supplemented data under the $(p+1) \times (p+1)$ pseudo-network. We use $\mathbf{\Sigma}_{p\times p}$ and $\tilde{\mathbf{\Sigma}}_{(p+1)\times(p+1)}$ to denote the corresponding covariance matrices. Note that,

$$Cov(\tilde{Y}_{j_1}, \tilde{Y}_{j_2}) = Cov(Y_{j_1} + a_{j_1}Z, Y_{j_2} + a_{j_2}Z) = Cov(Y_{j_1}, Y_{j_2}) + 2 \times 0 + a_{j_1}a_{j_2}Var(Z),$$
$$Cov(\tilde{Y}_j, Z) = Cov(Y_j + a_j Z, Z) = a_j Var(Z),$$

a_j demonstrates the marginal covariance between the pseudogene and the jth node in the pseudo-network. It can be shown that

$$\tilde{\mathbf{\Sigma}} = \begin{bmatrix} \mathbf{\Sigma} + \mathbf{a}\mathbf{a}' & \mathbf{a} \\ \mathbf{a}' & 1 \end{bmatrix}. \tag{1}$$

As $\mathbf{\Sigma} + \mathbf{a}\mathbf{a}' - \mathbf{a}\mathbf{a}'$ is indeed the original covariance matrix $\mathbf{\Sigma}$ and is always invertible, by the property of block matrix inverse,

$$\tilde{\mathbf{\Sigma}}^{-1} = \begin{bmatrix} \mathbf{\Sigma}^{-1} & -\mathbf{\Sigma}^{-1}\mathbf{a} \\ -\mathbf{a}'\mathbf{\Sigma}^{-1} & 1 + \mathbf{a}'\mathbf{\Sigma}^{-1}\mathbf{a} \end{bmatrix}, \tag{2}$$

where $\mathbf{a} = (a_1, ..., a_p)'$, $a_j \in R$, for $j = 1, ..., p$.

Equation 2 implies the first $p \times p$ block matrix in $\tilde{\mathbf{\Sigma}}^{-1}$ is exactly the original $\mathbf{\Sigma}^{-1}$. So, when we analyze the partial correlation graph of the supplemented data $\tilde{\mathbf{Y}}_i = (\tilde{Y}_{i,1}, \ldots, \tilde{Y}_{i,p}, Z_i)'$, the upper $p \times p$ block $\tilde{\mathbf{\Psi}}$ of the expanded partial correlation matrix of $\{\tilde{\mathbf{Y}}_i\}$ is the same as the partial correlation matrix of the original data. Furthermore, the block matrix $-\mathbf{a}'\mathbf{\Sigma}^{-1}$, denoted as $\mathbf{h}'$ (a $1 \times p$ row vector), characterizes the conditional correlations between the pseudogene Z and the original set of p genes. In practice, $\mathbf{h}'$ is unknown as $\mathbf{\Sigma}^{-1}$ is unknown. However, it is feasible to specify a sparse vector $\mathbf{a}$, then each component of $\mathbf{h}$ is a linear combination of only a few entries in $\mathbf{\Sigma}^{-1}$. Note that, non-zero a_j leads to non-zero h_j as the jth diagonal entry of $\mathbf{\Sigma}^{-1}$ is not zero. Moreover, since $\mathbf{\Sigma}^{-1}$ is assumed to be sparse, $\mathbf{h}$ is also a sparse vector that can be estimated precisely based on the original data. We consider estimating $\mathbf{\Sigma}^{-1}$ by Graphical Lasso (with R package glasso), denoted as $\hat{\mathbf{\Sigma}}^{-1}_{\text{glasso}}$, and then compute $\hat{\mathbf{h}}' = -\mathbf{a}'\hat{\mathbf{\Sigma}}^{-1}_{\text{glasso}}$. As $\mathbf{a}$ is designated to be sparse, the estimation error of $\hat{\mathbf{h}}'$ is expected to be small. Lastly, true connections between the pseudogene and the original set of genes are determined by non-zero a_js, because non-zero a_j leads to nonzero h_j. Nulls are estimated by $\hat{h}_j$, where $\hat{h}_j$ are ranked by their absolute value, and those of the lowest magnitudes are defined as "estimated nulls".

S-4.2 Application on *E. coli* Dataset

To assess the performance of different approaches on *E. coli* dataset, we randomly selected 20 out of 102 a_js, set as 0.1, while the rest were all 0. The set $\{j : a_j = 0.1, j = 1, 2, ..., 102\}$ was denoted as $\mathcal{L}$, $|\mathcal{L}| = 20$. Note $\mathcal{L}$ determines the true connections between the pseudogene and the original set of genes. To estimate null connections, Graphical Lasso was applied to the original dataset and the estimated precision matrix was denoted as $\hat{\mathbf{\Sigma}}^{-1}_{\text{glasso}}$. We then ranked $\hat{h}_j$ by its absolute value and considered the 20 of the lowest magnitude as "estimated nulls". For PCGII, 20 out of 20 true connections were incorporated as prior information because the knowledge is true and free to

use under the supplemented data analysis framework. To control variability arising from sampling Z_i, we fixed $\mathbf{a}$ and repeatedly generated supplemented dataset $\{\hat{\mathbf{Y}}_i\}^{(k)}$, $k = 1, 2, ..., K$. For each supplemented dataset $\{\hat{\mathbf{Y}}_i\}$ simulated, we applied four approaches. We stored the set of significant edges $\mathcal{S}_k$ found by each approach given FDR controlled at .05. The final results were aggregated from $\mathcal{S}_k$ by majority voting.

Supplementary Table S-1 shows the total discoveries, true discoveries of edges determined by non-zero a_js, and false discoveries of estimated null edges. PCGII identified 19 out of 20 true edges due to information incorporation, with a 0% error rate. The other three approaches all discovered some estimated null edges while identifying fewer true edges.

Venn diagrams in panels (a), (b), and (c) of Supplementary Figure S-10 showcase the intersections and distinctiveness of edges identified by CLEVEL, E-TEST, and F-GGM across both original and supplemented data analyses. Each diagram highlights the comparison between results obtained from the original and supplemented data analyses for each method. Panel (d) of Supplementary Figure S-10 is the Venn diagram displaying the relationship among three sets. Each of the three sets represents the reconstructed edges in the target *E. coli* network. Two sets depict the outcomes of supplemented data analysis using CLEVEL and PCGII, denoted as "CLEVEL on supplemented data" and "PCGII on supplemented data". The third set represents the results of the original data analysis by CLEVEL, serving as the reference. Four Venn diagrams suggest analysis of the original data without the pseudogene is highly consistent with the analysis of the supplemented dataset. This indicates that supplementing the observed data with the pseudogene did not seem to change the main results of the other methods.

Table S-1: Summary of Supplemented Data Analysis on *E. coli* Dataset.

**Method**	**# Total Discoveries**	**# True Discoveries**	**# False Discoveries**
PCGII	19	19	0
CLEVEL	7	3	2
E-TEST	10	3	2
F-GGM	13	2	4

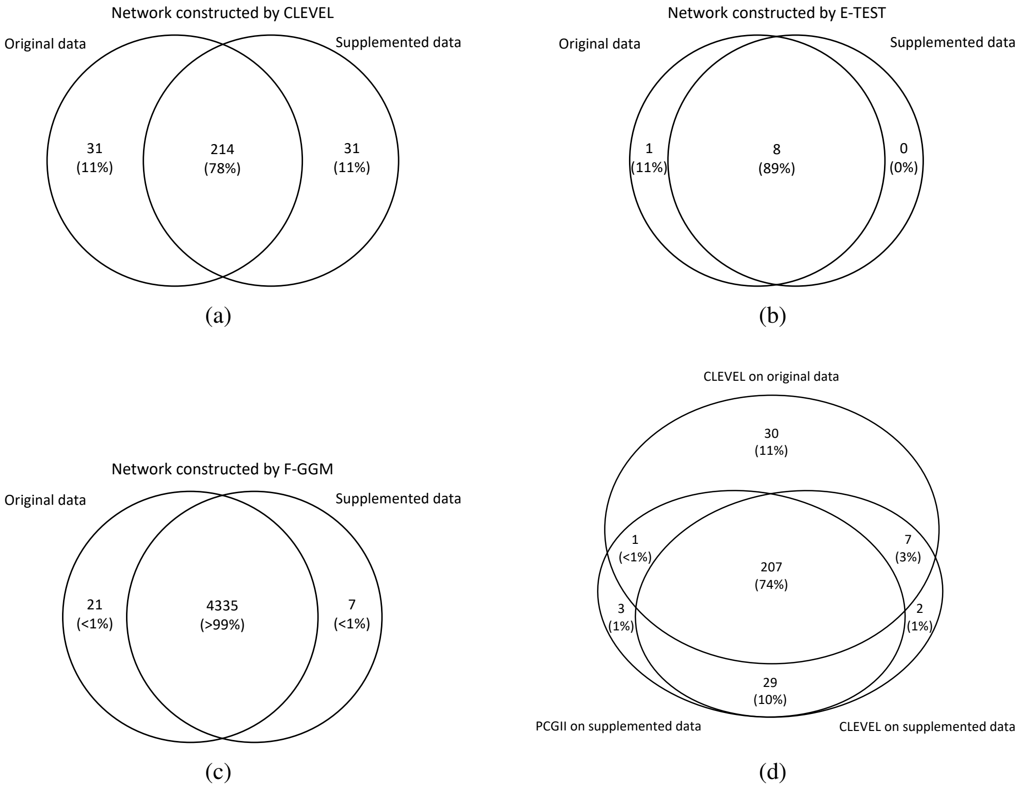

Figure S-10: Venn Diagrams of Reconstructed *E. coli* Networks.
Panels (a), (b), and (c) present Venn diagrams illustrating the intersections and unique edges identified by CLEVEL, E-TEST, and F-GGM across original and supplemented data analyses. Panel (d) shows the Venn diagram that compares reconstructed edges in the target *E. coli* gene network. The diagram depicts three sets: one containing the results of the original data analysis by CLEVEL (referred to as CLEVEL on original data), and the other two sets showcasing the outcomes of supplemented data analysis by CLEVEL and PCGII, respectively.

S-5 Supplementary Results for Sensitivity Analysis

In this section, we present the simulation setting and results of investigating how sensitive PCGII is concerning the size and accuracy of prior information. Supplementary Figures S-11, S-12, and S-13 present results for count data with moderate sample sizes under the scale-free structure, the random structure, and the block diagonal structure, respectively.

To further investigate how PIS and PA affect the performance of PCGII, we considered a 3 by 3 factorial design additionally, where PIS $= 100\%, 70\%, 30\%$ and PA $= 1, 0.7, 0.3$. The power and empirical FDR of PCGII were computed by averaging across 500 datasets.

Note the two prior information scenarios, one with PIS=100% and PA=0.7 and the other with PIS=70% and PA=1, both provide 70% of true edges. The distinction lies in the nature of the prior information utilized. In the first case, the prior information contained 30% false information, whereas in the second case, only true information was incorporated. Comparing the results from these two applications will shed light on the impact of including false positives in the prior set of the proposed method.

The findings indicate that the higher the proportion of correct information in the prior, the greater the power of PCGII. Whether the prior set contains false edges or not, the ROC curves and power are quite similar as long as an equivalent amount of correct information is provided to PCGII. For instance, the ROC curves from the settings with PIS=100% and PA=0.7, and PIS=70% and PA=1, exhibit substantial overlap, and the box plots of total power for both settings show comparable distributions.

When the prior information is accurate, FDR is effectively controlled regardless of the prior information size. However, inaccurate prior information inflates the empirical FDR, and the extent of inflation is influenced by the amount of false information included. When the prior information is inaccurate (e.g., PA=0.3), PCGII does not solely rely on the prior information but also leverages the data to recover the network. Notably, a network constructed purely based on the prior information with PA=0.3 results in a false discovery proportion of 0.7. In such instances, PCGII improves the network with a much lower FDR of approximately 0.3, indicating its capability to fully use the data for network recovery.

Furthermore, larger sample sizes enhance power and FDR control, whereas the number of nodes and the complexity of the network do not significantly impact the results. These findings are consistent across various network settings.

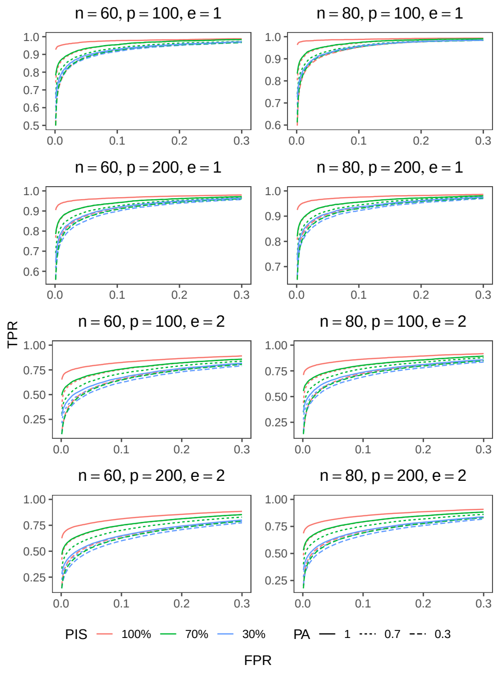

(a)

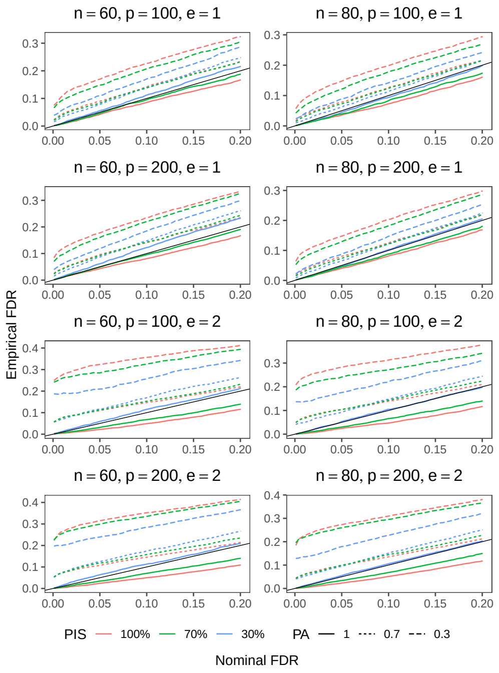

(b)

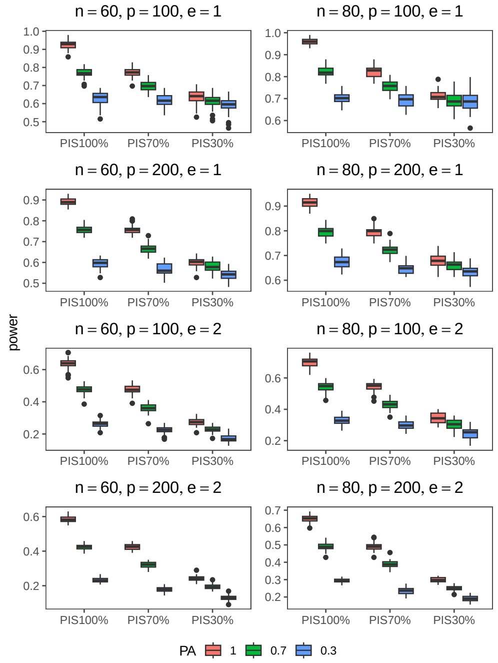

(c)

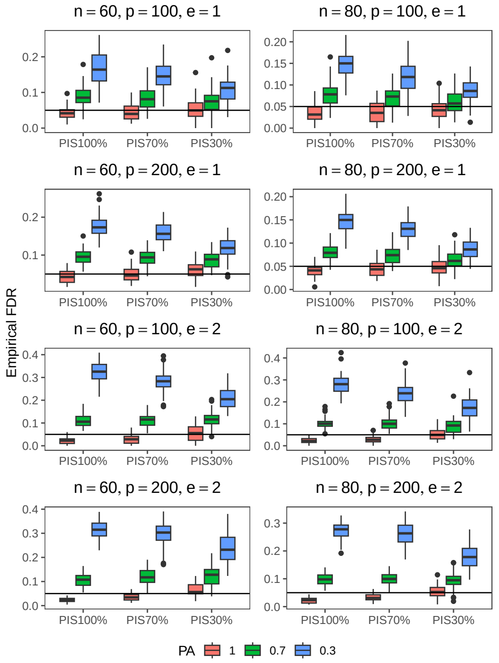

(d)

Figure S-11: Performance of PCGII across Different Settings of Prior Information under The Scale-free Structure.
(a) ROC curve; (b) empirical FDR; (c) box-plots of power across 500 datasets for each setting while FDR was controlled at the nominal level of 0.10; (d) box-plots of empirical FDR across 500 datasets for each setting while FDR was controlled at the nominal level of 0.10. PIS: prior information size; PA: prior accuracy.

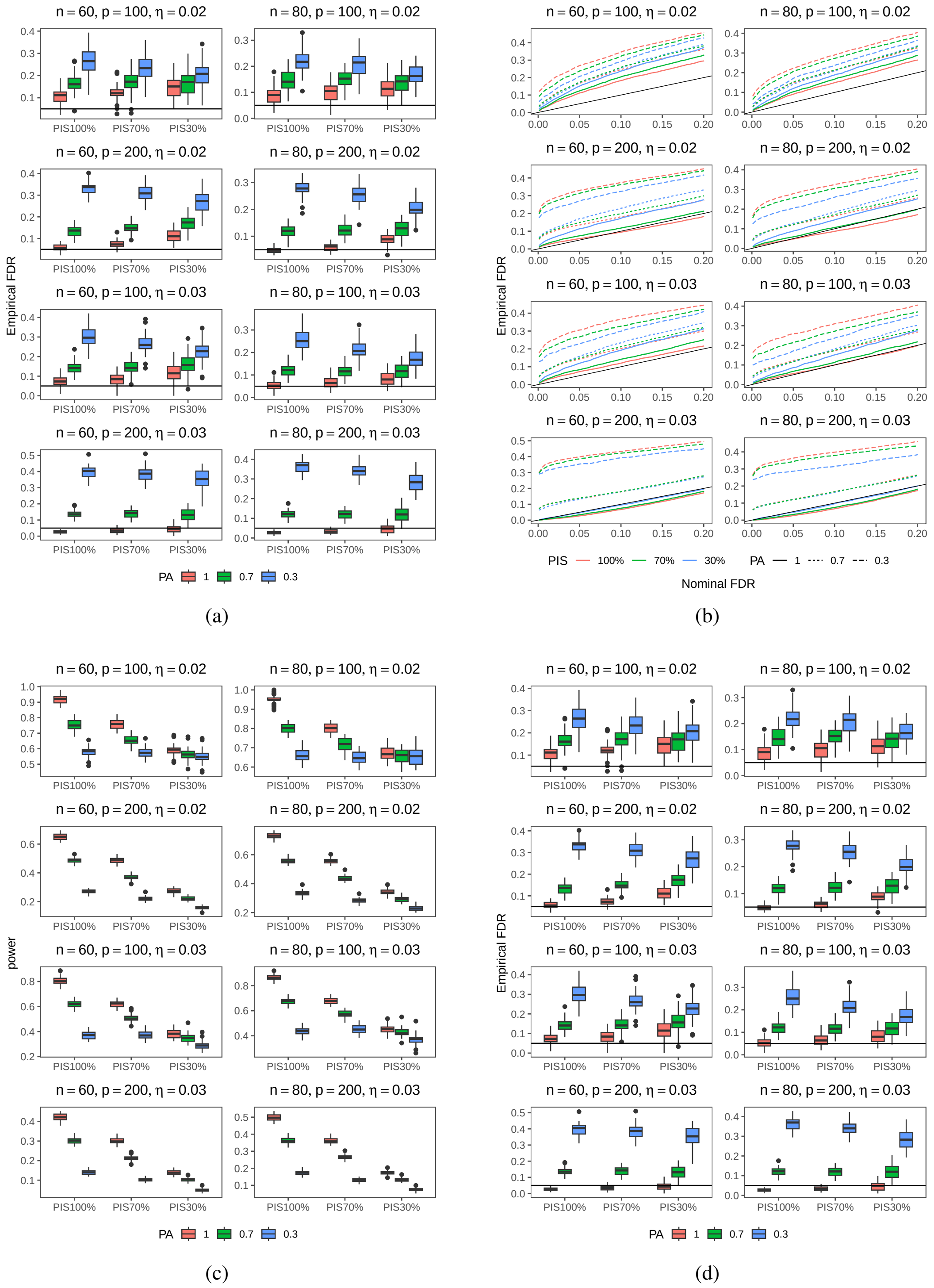

Figure S-12: Performance of PCGII across Different Settings of Prior Information under The Random Structure.

(a) ROC curve; (b) empirical FDR; (c) box-plots of power across 500 datasets for each setting while FDR was controlled at the nominal level of 0.10; (d) box-plots of empirical FDR across 500 datasets for each setting while FDR was controlled at the nominal level of 0.10. PIS: prior information size; PA: prior accuracy.

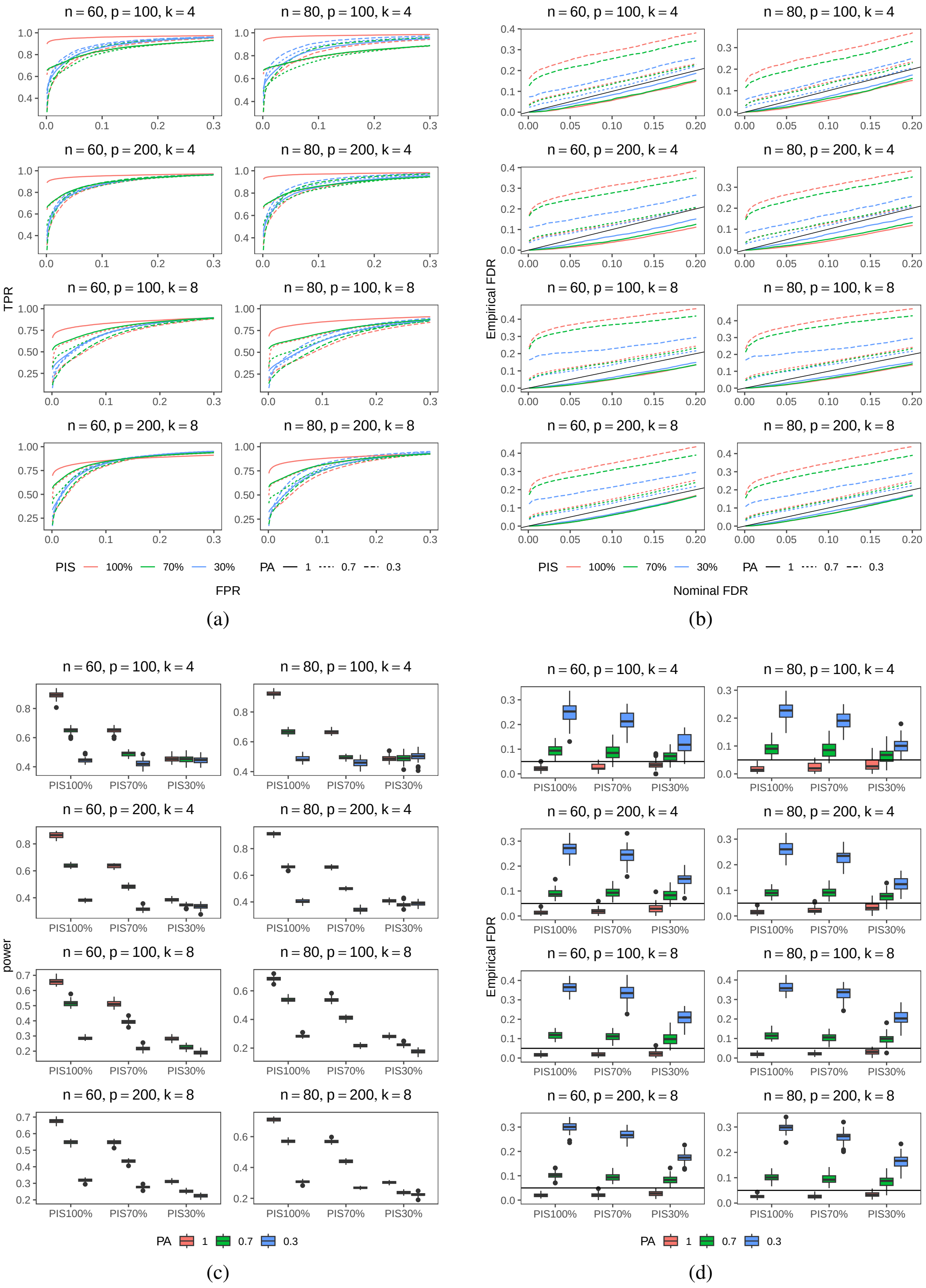

Figure S-13: Performance of PCGII across Different Settings of Prior Information under The Block Diagonal Structure.

(a) ROC curve; (b) empirical FDR; (c) box-plots of power across 500 datasets for each setting while FDR was controlled at the nominal level of 0.10; (d) box-plots of empirical FDR across 500 datasets for each setting while FDR was controlled at the nominal level of 0.10. PIS: prior information size; PA: prior accuracy.
